# High IgA antiphospholipid autoantibodies in healthy Sudanese explain the increased prevalence among Sudanese compared to Swedish systemic lupus erythematosus patients

**DOI:** 10.1177/0961203320945387

**Published:** 2020-08-02

**Authors:** Sahwa Elbagir, Amir I Elshafie, Elnour M Elagib, NasrEldeen A Mohammed, Mawahib IE Aledrissy, Vivek Anand Manivel, Eleftheria Pertsinidou, Musa AM Nur, Iva Gunnarsson, Elisabet Svenungsson, Johan Rönnelid

**Affiliations:** 1 Department of Immunology, Genetics and Pathology, Uppsala University, Uppsala, Sweden; 2 Rheumatology Unit, Alribat University Hospital, Khartoum, Sudan; 3 Rheumatology Unit, Military Hospital, Omdurman, Sudan; 4 Faculty of Medical Laboratory Sciences, Al Neelain University, Khartoum, Sudan; 5 Division of Rheumatology, Department of Medicine Solna, Karolinska Institutet, Karolinska University Hospital, Stockholm, Sweden

**Keywords:** Systemic lupus erythematosus, Sudan, Africa, Sweden, antiphospholipid antibodies, IgA, anti-β2-glycoprotein I

## Abstract

**Objectives:**

IgA antiphospholipid antibodies (aPL) are prevalent in systemic lupus erythematosus (SLE) patients of African American, Afro-Caribbean and South African origin. Nevertheless, data from North Africa are lacking, and most studies use manufacturer-suggested cut-offs based on Caucasian controls. Therefore, we compared aPL isotypes in Sudanese and Swedish SLE patients using nation-based cut-offs.

**Methods:**

Consecutive SLE patients and age- and sex-matched controls from Sudan (*N* = 115/106) and Sweden (*N* = 340/318) were included. All patients fulfilled the 1982 American College of Rheumatology SLE classification criteria. Antiphospholipid syndrome–related events were obtained from patients’ records. IgA/G/M anticardiolipin and anti-β_2_ glycoprotein I (β_2_GPI) were analysed with two independent assays. IgA anti-β_2_GPI domain 1 (D1) was also investigated. Manufacturers’ cut-offs and the 95th and 99th percentile cut-offs based on national controls were used.

**Results:**

Sudanese patients and controls had higher levels and were more often positive for IgA aPL than Swedes when using manufacturers’ cut-offs. In contrast, using national cut-offs, the increase in IgA aPL among Sudanese patients was lost. Occurrence of IgA anti-D1 did not differ between the countries. Venous thromboses were less common among Sudanese patients and did not associate with aPL. No clinical associations were observed with IgA anti-β_2_GPI in Sudanese patients. Thromboses in Swedes were associated with IgG/M aPL. Fetal loss was associated with aPL in both cohorts.

**Conclusions:**

IgA anti-β_2_GPI prevalence was higher among Sudanese compared to Swedish patients when manufacturers’ cut-offs were used. This situation was reversed when applying national cut-offs. Anti-D1 was not increased in Sudanese patients. Previous studies on populations of African origin, which demonstrate a high prevalence of IgA aPL positivity, should be re-evaluated using a similar cut-off approach.

## Introduction

Antiphospholipid antibodies (aPL) are present in 20–30% of systemic lupus erythematosus (SLE) patients,^1^ where IgA anti-β_2_ glycoprotein I (β_2_GPI) is reported to be a highly frequent isotype.^2–6^ Only IgG and IgM anticardiolipin (aCL) and anti-β_2_GPI are included in the 2006 revised Sapporo classification criteria for antiphospholipid syndrome (APS),^7^ whereas IgA aPL were added as laboratory parameters to the 2012 Systemic Lupus International Collaborating Clinics (SLICC)/American College of Rheumatology (ACR)^8^ as well as the recent European League Against Rheumatism EULAR/ACR SLE classification criteria.^9^ The role of IgA aPL as a risk factor for thrombosis and adverse pregnancy outcomes in SLE remains inconclusive. A positive association with APS-related events was demonstrated in several studies for both IgA aCL^2^,^3^,^10^ as well as IgA anti-β_2_GPI.^2^,^4^,^11–13^ However, other investigators could not confirm these associations.^3^,^10^,^14^,^15^ Interpretation of data provided by previous studies on aPL in general and IgA aPL in particular and the relationship to APS features is rather complex. The fact that different study designs, immunological assays and cut-offs were used in these studies imposes more challenges in determining the true prevalence and clinical significance of aPL among SLE patients in different populations. Insufficient standardization of IgA aPL assays contributes to this enigma, heterogeneity was evident when comparing different diagnostic kits.^16–18^ In addition, the frequent co-occurrence of other isotypes with IgA makes it even more troublesome to ascertain the individual role of IgA aPL in SLE.

Data are limited with regard to the prevalence of aPL isotypes, including IgA, and their clinical importance among SLE populations of African origin, with utterly deficient studies among populations living in Africa. According to the current literature, IgA aPL are described to be the most prevalent and clinically significant isotypes in SLE patients of African ancestry. Previous studies reported highly prevalent IgA anti-β_2_GPI among African American SLE patients with associations with thrombosis and/or other APS-related events.^2^,^4^,^19^ In an earlier study of 100 black South African SLE patients, IgA aCL was found to be the most prevalent isotype, where both IgA aCL and IgA anti-β_2_GPI were significantly associated with thrombosis.^20^ In all of these studies conducted in populations of African origin, determination of cut-offs for aPL positivity was based on the assay manufacturers’ recommendation, or on values calculated according to levels in healthy controls using parametric statistical methods but where characteristics of the reference groups were not specified. Since distribution of aPL is not Gaussian, it is generally recommended to calculate aPL cut-offs using non-parametric statistics.^7^,^21^

The aim of this study was to determine the prevalence and clinical significance of the different aPL isotypes, with special emphasis on IgA. We also investigated IgA anti-β_2_GPI domain 1 (anti-D1) using conventional as well as nationally adjusted non-parametric cut-offs.

## Methods

### Participants

This cross-sectional study included 115 Sudanese and 340 Swedish SLE patients. Serum samples were available from 93 Sudanese and 333 and Swedish SLE patients as well as from 106 healthy and 297 population-based non-SLE controls, respectively. All controls were matched for age and sex to the respective patient groups. The lack of serum from some Sudanese patients was entirely due to technical reasons without selection bias. All SLE patients fulfilled the 1982 revised (ACR) criteria.^22^ A detailed description of cohorts was specified in our previous publication.^23^ Clinical comparisons were first performed between the full cohorts. Due to the highly significant difference in age and disease duration, where Swedish patients were older with longer SLE duration, comparisons were also performed between nested cohorts matched for age and disease duration, including 88 Sudanese and 88 Swedish patients, in agreement with our previous study.^23^ Clinical information including APS-related events was obtained from patients’ hospital records. These included a history of thrombotic events (venous: deep venous thrombosis and pulmonary embolism; and arterial: cerebrovascular accidents, myocardial infarction and peripheral tissue loss as described in the SLICC Damage Index (SDI)),^24^ obstetric events (early and late miscarriage, intrauterine fetal demise (IUFD) and stillbirths) and cardiovascular (CVS) events as defined by the SDI.^24^ The occurrence of thrombocytopaenia at study inclusion for Swedish patients and a history of immune thrombocytopaenic purpura (ITP) for Sudanese patients were also investigated for aPL occurrence.

All participants gave written informed consent, and their inclusion was in accordance with the Declaration of Helsinki. Approval was obtained from Ethical Committees of the study recruitment hospitals in Sudan (11 April 2011 and 25 May 2011) and Sweden (03-556; 16 December 2003).

### Immunological testing

Quantification of aCL and anti-β_2_GPI isotypes was performed using two assays: the EliA system based on fluorescence enzyme immunoassay (FEIA) on a Phadia 2500 instrument (Thermo Fisher Scientific, Uppsala, Sweden) according to the manufacturer’s instructions, and the Aptiva system based on a particle-based multi-analyte technology (PMAT; Inova Diagnostics, San Diego, CA). IgA anti-D1 was analysed using modified a QUANTA Flash β_2_GPI-D1 chemiluminescence assay (BIO-FLASH; Inova Diagnostics). Specific analysis of the IgA isotype against D1 was performed in parallel with a previous study where a high prevalence of IgA anti-β_2_GPI was observed during apparently normal pregnancies in Sudan, as previously described.^25^ Lupus anticoagulant (LA) was not included in this study due to difficulties in conforming to sample handling recommendations for LA testing in Sudanese SLE patients.^26^

FEIA analyses were performed on different occasions in Sudanese and Swedish subjects, whereas PMAT and IgA anti-D1 analyses were performed in parallel. However, in all cases, analyses of patients and national controls were performed in parallel.

The number of patients who had laboratory data for each immunological assay is shown in Table 2. All controls from both countries had aPL results using the FEIA, while only 102 Sudanese and 163 Swedish subjects had available aPL data using PMAT.

### Statistical analysis

For comparisons between quantitative variables, the Mann–Whitney *U*-test was used, whereas the chi-square test was used to test for differences between categorical variables, with Fisher’s exact test applied when appropriate.

Three cut-offs were identified: the manufacturers’ suggested values (same for Sudanese and Swedish patients) and the 95th and 99th percentiles of the respective national controls. Non-parametric statistics were used to identify national cut-offs, since all aPL levels, irrespective of assay used, were not normally distributed, as tested by the Shapiro–Wilk test in controls (data not shown). The 99th national cut-offs were not used in the clinical association analyses, as the small number of Sudanese controls makes this cut-off uncertain. Data comparing the prevalence of aPL using the 99th percentile are shown with and without the exclusion of outliers using the modified Dixon D/R method by Reed.^27^,^28^

All statistical analyses including cut-off determination were conducted using JMP (SAS Institute, Cary, NC). *p-*Values <0.05 were considered significant.

## Results

### Demographics and clinical comparisons

Comparing APS-related events in the full cohorts, Sudanese SLE patients had a lower prevalence of venous thrombosis (7% vs, 15%; *p* = 0.03) compared to Swedish patients (Table 1). In agreement with our previous publication, Sudanese patients had a higher female preponderance and shorter disease duration compared to Swedish patients.^23^ In the cohorts matched for age and disease duration, the occurrence of thrombosis or fetal loss did not differ between Sudanese and Swedish patients (Table 1).

**Table 1.** Demographics and APS-related events in the investigated SLE patients.

	Sudan all patients (*N* = 115)	Sweden all patients (*N* = 340)	*p*-Value	Sudan matched patients (*N* = 88)	Sweden matched patients (*N* = 88)	*p*-Value
Sex (F/M)	110/5	297/40	**0.02**	85/3	79/9	0.07
Age at inclusion (years), median/mean	33/34.9	47.7/46.7	**<0.0001**	35/36.8	35.4/37.3	0.7
Any thrombosis	15 (13%)	77 (23.2%)	**0.02**	13 (14.8%)	14 (15.9%)	0.8
Venous thrombosis	8 (7%)	50 (15%)	**0.03**	7 (8%)	13 (14.8%)	0.1
Arterial thrombosis	10 (8.7%)	37 (11.1%)	0.5	9 (10.2%)	3 (3.4%)	0.07
Any obstetric event	37 (34.3%)	71 (26.9%)	0.1	31 (36.9%)	16 (23.2%)	0.06
Early miscarriage	19 (17%)	44 (15.8%)	0.8	18 (20.9%)	10 (13.5%)	0.2
Late miscarriage	18 (16.1%)	37 (13.2%)	0.5	14 (16.3%)	9 (12.2%)	0.4
IUFD/stillbirths	4 (3.7%)	7 (2.7%)	0.6	3 (3.6%)	1 (1.5%)	0.4
CVS	7 (6.1%)	33 (9.8%)	0.2	5 (5.7%)	1 (1.1%)	0.2

Sudanese and Swedish patients are compared both concerning the full cohorts as well as the nested group matched for age and disease duration. Significant differences are depicted in bold.

APS: antiphospholipid syndrome; SLE: systemic lupus erythematosus; IUFD: intrauterine fetal demise; CVS: cardiovascular.

### aPL profile

**Figure 1.**
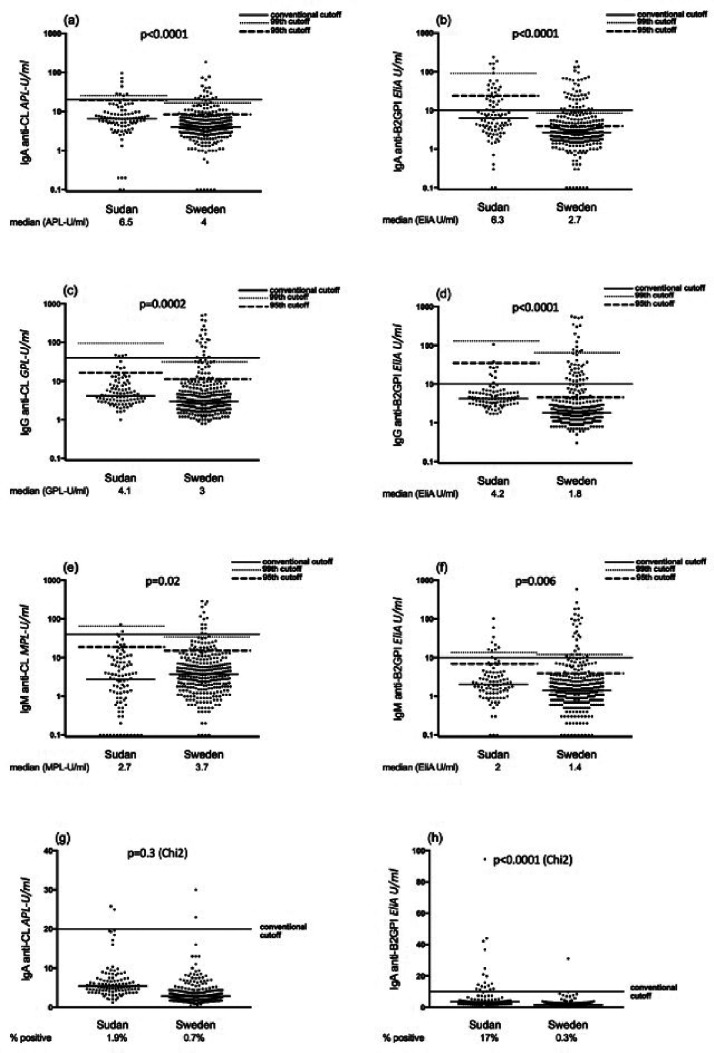
Levels of IgA/G/M anticardiolipin (aCL) and anti-β_2_ glycoprotein I (β_2_GPI) analysed with fluorescence enzyme immunoassay among systemic lupus erythematosus (SLE) patients and controls. (a), (c) and (e) IgA/G/M aCL, and (b), (d) and (f) IgA/G/M anti-β_2_GPI levels among Sudanese and Swedish SLE patients. Horizontal lines represent manufacturers’ cut-offs, the 95th and 99th percentile cut-off values of national controls, without exclusion of outliers. (g) and (h) IgA aCL and IgA anti-β_2_GPI levels among Sudanese and Swedish controls. *p*-Values represent comparisons using the Mann–Whitney *U*-test for levels between patients ((a)–(f)) and the chi-square test for percentages between controls ((g) and (h)).

**Figure 2.**
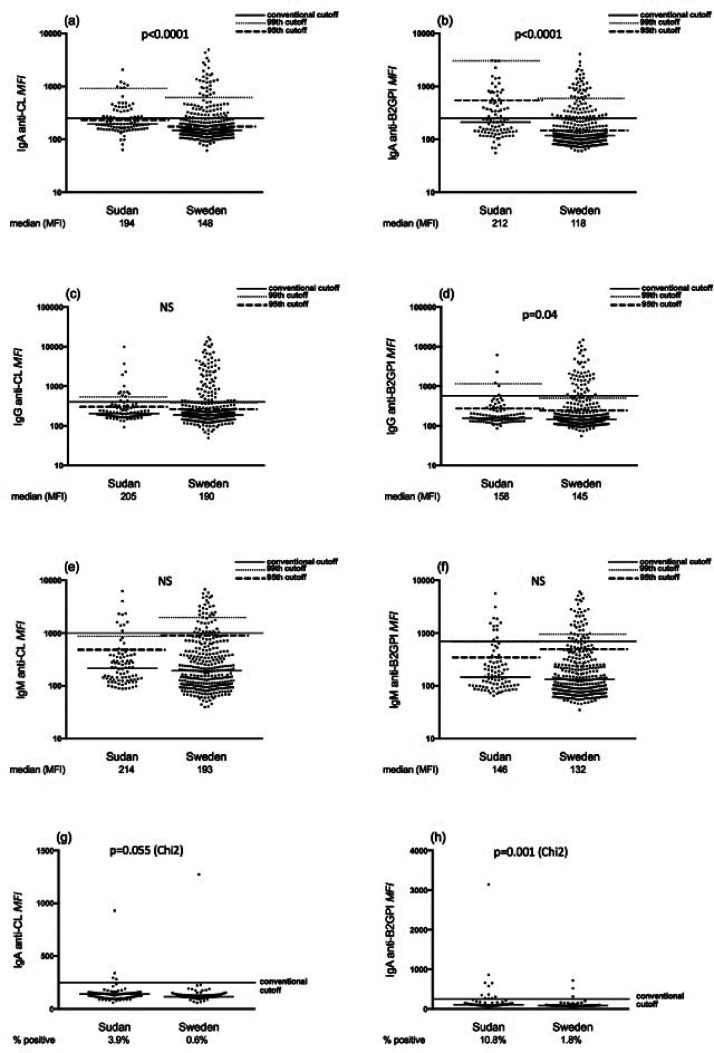
Levels of IgA/G/M aCL and anti-β_2_GPI analysed with particle-based multi-analyte technology among SLE patients and controls. (a), (c) and (e) IgA/G/M aCL and (b), (d) and (f) IgA/G/M anti-β_2_GPI levels among Sudanese and Swedish SLE patients. Horizontal lines represent manufacturers’ cut-offs, the 95th and 99th percentile cut-off values of national controls, without exclusion of outliers. (g) and (h) IgA aCL and IgA anti-β_2_GPI levels among Sudanese and Swedish controls. *p*-Values represent comparisons using the Mann–Whitney *U*-test for levels between patients ((a)–(f)), and chi-square test for percentages between controls ((g) and (h)).

Sudanese SLE patients had higher levels of IgA and IgG aPL compared to Swedish patients. This finding was replicated with both assays, as depicted in Figures 1 and 2 and Table 2. Sudanese patients also had a significantly higher prevalence of IgA anti-β_2_GPI using assay manufacturers’ suggested cut-offs common to both countries (FEIA: 34.8% vs. 14.9%, *p*<0.0001; PMAT 41.8% vs. 23%, *p* = 0.0004). Using the same cut-offs, IgA anti-β_2_GPI was the most common antibody among SLE patients in both Sudan and Sweden (Table 2). Comparing control subjects from Sudan and Sweden, levels of IgA/IgG aCL and anti-β_2_GPI were significantly higher among Sudanese subjects, irrespective of the assay used (Table 2). The percentage of controls testing positive for IgA anti-β_2_GPI using manufacturers’ cut-offs was significantly higher among Sudanese compared to Swedish subjects (Figures 1(h) and 2(h)). By adjusting cut-offs to the 95th and 99th percentiles of national controls, this picture shifted so that Swedish patients now had a higher prevalence of IgA anti-β_2_GPI, irrespective of assay used (Table 2). Also, using the calculated 99th national cut-off after exclusion of outlier values among controls, the increased prevalence of IgA aPL in Sudanese patients was lost. However, Sudanese patients showed higher IgM aPL positivity compared to Swedish patients (Table 2).

**Table 2.** Levels and prevalence of aCL and anti-β_2_GPI isotypes among Sudanese and Swedish patients and controls.

	FEIA	FEIA		PMAT/CIA	PMAT/CIA	
aPL in SLE patients	Sudan (*N* = 92)	Sweden (*N* = 311)	*p*-Value	Sudan (*N* = 93)	Sweden (*N* = 333)	*p*-Value
IgA CL, median/mean	6.5/10.3	4/7.2	**<0.0001**	194/304.9	148/315.3	**<0.0001**
IgG CL, median/mean	4.1/7.8	3/16.6	**0.0002**	205/442.9	190/873.8	0.09
IgM CL, median/mean	2.7/6.7	3.7/11.1	**0.02**	214/482.9	193/544.7	0.2
IgA β_2_GPI, median/mean	6.3/20.8	2.7/8.8	**<0.0001**	212/452.3	118/268.2	**<0.0001**
IgG β_2_GPI, median/mean	4.2/7.1	1.8/16.9	**<0.0001**	158/305.2	145.2/613	**0.04**
IgM β_2_GPI, median/mean	2.0/5.6	1.4/9.6	**0.006**	146.2/406.6	132.5/425	0.09
β2GP1 D1 IgA, median/mean				1542/2301	1683/5147	**0.045**
IgA CL common cut-off	9 (9.8)	17 (5.8)	0.2	32 (35.2)	75 (22.7)	**0.02**
IgG CL common cut-off	4 (4.3)	21 (6.7)	0.4	14 (15)	72 (22.1)	0.1
IgM CL common cut-off	3 (3.2)	14 (4.5)	0.6	10 (11.4)	43 (13.1)	0.6
IgA β_2_GPI common cut-off	32 (34.8)	43 (14.9)	**<0.0001**	38 (41.8)	76 (23)	**0.0004**
IgG β_2_GPI common cut-off	10 (10.9)	44 (14.1)	0.4	5 (5.4)	50 (15.3)	**0.01**
IgM β_2_GPI common cut-off	10 (10.9)	27 (8.7)	0.5	12 (13.6)	42 (12.9)	0.8
IgA β2GP1 DI common cut-off				6 (6.4)	38 (11.4)	0.2
IgA CL 95th cut-off	9 (9.8)	54 (18.6)	**0.047**	36 (39.6)	121 (26.6)	0.6
IgG CL 95th cut-off	8 (8.7)	42 (13.5)	0.2	24 (25.8)	103 (31.6)	0.3
IgM CL 95th cut-off	8 (8.7)	39 (12.5)	0.3	16 (18.2)	47 (14.4)	0.4
IgA β_2_GPI 95th cut-off	18 (19.6)	100 (34.72)	**0.006**	20 (22)	125 (37.8)	**0.005**
IgG β_2_GPI 95th cut-off	2 (2.1)	67 (21.5)	**<0.0001**	19 (20.4)	83 (25.5)	0.3
IgM β_2_GPI 95th cut-off	14 (15.22)	57 (18.3)	0.5	20 (22.7)	57 (17.5)	0.3
IgA β2GP1 D1 95th cut-off				25 (26.9)	69 (20.7)	0.2
IgA CL 99th cut-off	7 (7.6)	21 (7.2)	0.9	7 (7.7)	31 (9.4)	0.6
IgG CL 99th cut-off	0 (0)	25 (8.0)	**0.005**	12 (12.9)	75 (23)	**0.03**
IgM CL 99th cut-off	1 (1.1)	15 (4.8)	0.1	10 (11.4)	24 (7.3)	0.2
IgA β_2_GPI 99th cut-off	6 (6.5)	50 (17.4)	**0.01**	1 (1.1)	35 (10.6)	**0.004**
IgG β_2_GPI 99th cut-off	0 (0)	15 (4.8)	**0.03**	3 (3.2)	56 (17.2)	**0.0006**
IgM β_2_GPI 99th cut-off	9 (9.8)	22 (7.1)	0.4	12 (13.6)	33 (10.1)	0.3
IgA β2GP1 D1 99th cut-off				6 (6.4)	28 (8.4)	0.5
IgA CL 99th cut-off without outliers	7 (7.6)	21 (7.2)	0.9	23 (25.3)	86 (26)	0.8
IgG CL 99th cut-off without outliers	4 (4.3)	24 (7.9)	0.2	12 (12.9)	75 (23)	**0.03**
IgM CL 99th cut-off without outliers	1 (1.1)	15 (4.8)	0.1	13 (14.8)	24 (7.3)	**0.03**
IgA β_2_GPI 99th cut-off without outliers	10 (10.9)	53 (18.4)	0.09	11 (12.1)	35 (10.6)	0.6
IgG β_2_GPI 99th cut-off without outliers	1 (1.1)	17 (5.4)	0.07	19 (20.4)	65 (19.9)	0.9
IgM β_2_GPI 99th cut-off without outliers	9 (9.8)	22 (7.1)	0.4	27 (30.6)	57 (17.5)	**0.006**
aPL in controls	Sudan (*N* = 106)	Sweden (*N* = 297)	*p*-Value	Sudan (*N* = 102)	Sweden (*N* = 163)	*p*-Value
IgA CL, median/mean	5.4/6.2	2.8/3.4	**<0.0001**	140/144	116/126	**<0.0001**
IgG CL, median/mean	4.7/7.2	2.8/4.2	**<0.0001**	170/184	157/166	**0.01**
IgM CL, median/mean	3.1/5.4	2.2/4.1	0.06	156/196	193/324	**0.004**
IgA β_2_GPI, median/mean	3.4/6.8	1.5/1.9	**<0.0001**	105/169	83/94	**<0.0001**
IgG β_2_GPI, median/mean	4.5/8.4	1.3/3	**<0.0001**	132/158	120/137.4	**0.01**
IgM β_2_GPI, median/mean	2.3/2.6	0.7/1.2	**<0.0001**	100/130	114/177	0.1
IgA β2GP1 D1, median/mean				1132/1300	1439/1660	**<0.0001**

Occurrence of IgA/IgG/IgM aCL and anti-β_2_GPI (*n* (%)) compared using three cut-offs: values recommended by the manufacturers and separate cut-offs based on the 95th and 99th percentiles among national controls. aCL and β_2_GPI isotypes are measure with both FEIA and PMAT, and IgA anti-D1 was analysed with CIA; levels expressed as median/mean. Number of serum samples available for each assay is shown in the respective columns. Significant *p*-values are depicted in bold.

aCL: anticardiolipin antibodies; aPL: antiphospholipid antibodies; β_2_ GPI: β_2_ glycoprotein I; FEIA: fluorescence enzyme immunoassay; PMAT: particle-based multi-analyte technology; CIA: chemiluminescence assay; D1; domain 1.

In contrast to IgA anti-β_2_GPI, both Swedish patients and controls exhibited higher levels of IgA anti-D1 compared to the corresponding Sudanese groups. The prevalence of IgA anti-D1 did not differ between Sudanese and Swedish patients, regardless of cut-offs (Table 2).

Levels obtained with the FEIA and PMAT correlated strongly for all investigated aPL and with similar figures for Sudanese and Swedish patients (Spearman’s ρ = 0.47–0.83 for all patients). For each immunoglobulin isotype, the degree of correlation was always stronger for anti-β_2_GPI than for aCL, and for each specificity, the degree of correlation was highest for IgA aPL followed by IgM and IgG. The highest degree of correlation was found for IgA anti-β_2_GPI (ρ = 0.83).

### Clinical associations with aPL

#### Sudanese patients

None of the aPL associated with venous or arterial thrombotic events, whereas CVS events associated with IgG aCL (*p* = 0.04) and IgM anti-β_2_GPI (*p* = 0.01). Early miscarriage was more prevalent among IgM aCL and IgM anti-β_2_GPI-positive patients (*p* = 0.005 and *p* = 0.03), while a history of IUFD/stillbirths was only associated with IgM anti-β_2_GPI (*p* = 0.04). A history of ITP was more common in IgA anti-D1 positive compared to negative patients (*p* = 0.004). Patients with thrombocytopaenia at study inclusion were few (*n* = 2), and no associations to any aPL were observed (data not shown). Adjusting aPL cut-offs to national controls increased autoantibody associations to adverse pregnancy outcomes and exclusively showed an association with ITP (Supplemental Table S1).

#### Swedish patients

In contrast to Sudanese patients, aPL in general were associated with thrombotic events in the full cohorts (Supplemental Table S2). Venous thrombosis was more common among patients positive for most measures of IgG aPL and many measures of IgA aPL, including IgA anti-D1 autoantibodies. On the other hand, arterial thrombosis was associated only with IgA anti-D1 (*p* = 0.008). IgG aCL were associated with early and late miscarriages as well as cardiovascular events (*p* = 0.03, *p* = 0.02 and *p* = 0.03, respectively), whereas IgA isotypes of all aPL were associated with stillbirth. Thrombocytopaenia was more common in IgA/IgG aCL and anti-β_2_GPI-positive patients. In contrast to evaluations using manufacturers’ cut-offs, adjustment to the 95th national percentile showed positive aPL associations with miscarriage and stillbirths (Supplemental Table S2). In the matched Swedish cohort where the number of patients was much lower, most aPL associations with thromboses were lost and remained only for venous thrombosis and IgG aCL (*p* = 0.02) and IgG anti-β_2_GPI (*p* = 0.048; data not shown).

## Discussion

In this study, we investigated and compared the prevalence and clinical significance of aCL and β_2_GPI isotypes among SLE patients from Sudan and Sweden applying analogous methodology including assays and cut-offs used.

We detected that high IgA aPL levels and positivity was common among Sudanese patients using manufacturers’ cut-offs. Intriguingly, levels were also higher among Sudanese compared to Swedish controls, and when using nationally adjusted cut-offs, this difference disappeared. In some comparisons, Swedish patients instead showed an increased prevalence of IgA aPL, most evident for IgA anti-β_2_GPI.

It is consistently reported that IgA anti-β_2_GPI antibodies are the most commonly occurring aPL in SLE patients with African ancestry.^2^,^4^,^19^,^29^ In contrast to our current study methodology, all previous studies on aPL in SLE among African populations have either used common manufacturers’ cut-offs, most probably based on Caucasian controls, or have adjusted cut-offs based on parametric statistics assuming normal distribution of aPL levels. As our data were non-normally distributed, and as non-parametric determination of aPL cut-offs was recommended by the latest Sapporo criteria for APS diagnosis^7^ as well as the International Task Force on the 14th aPL Congress on Laboratory Diagnostics and Trends,^21^ we regard our approach of adjusting cut-offs to age- and sex-matched national controls as a strength of the current study. Indeed, this approach revealed a different picture compared to commonly used manufacturers’ cut-offs (Table 2).

In our recently published paper on 120 healthy pregnant women from Sudan, markedly increased IgA aPL levels and prevalence were observed compared to non-pregnant controls. This finding was not seen in Swedish healthy pregnancies and was not accompanied by a parallel increase in IgA anti-D1.^25^ Moreover, in a former study on 89 Tunisian patients with inflammatory bowel diseases, a high prevalence of IgA anti-β_2_GPI, adjusted for total IgA, was reported using the 95th percentile of national controls.^30^ A similarly high prevalence of IgA anti-β_2_GPI was also reported in primary biliary cirrhosis patients from the same country.^31^ Therefore, from these studies, our current study and previously published data, we speculate that increased occurrence of IgA aPL, most clearly shown for anti-β_2_GPI, may be a propensity not only in Sudan but also in other regions of North Africa. We believe that the increased level of IgA aPL is not limited to SLE patients but rather is a general phenomenon seen in both healthy and diseased Sudanese subjects. Detection of IgA aPL has also been reported in non-APS conditions (e.g. metabolic, renal and infectious diseases).^32–34^ An association with infections was shown since the early description of aCL antibodies.^35^,^36^ Given the fact that infection is also considered as one of the possible second hits triggering APS, it is difficult to consider infection-induced aPL as totally innocent. The increased IgA aPL levels in African populations might reflect mucosal immunity and/or molecular cross-reactivity with infectious agents. Conventionally speaking, infection-related aPL should mostly react with the cardiolipin moiety (and not with the added co-factors), but an increase in anti-β_2_GPI was also reported in South African patients with viral and parasitic infections.^37^ Therefore, further investigations of antibodies against individual domains of β_2_GPI or other pathogenic ‘non-criteria’ aPL could be of value in these populations.

Occurrence of IgA anti-D1 antibodies did not differ between Sudanese and Swedish SLE patients, and thus the increased IgA reactivity to β_2_GPI in Sudanese individuals was not primarily against the D1 epitope. Our current data are, on the other hand, in agreement with our recent report showing an increase in IgA anti-β_2_GPI but not in IgA anti-D1 among healthy pregnant women in Sudan.^25^ IgG anti-D1 is consistently reported to be accountable for APS-related events.^38^,^39^ However, data on the significance of IgA/IgM isotypes are deficient. Despierres et al. previously showed that IgA anti-domain 4/5 and not IgA anti-D1 are associated with SLE.^40^ In a recent study by Serrano et al., APS patients with thrombosis more often had IgA antibodies against β_2_GPI domains 3, 4 and 5 than they had IgA anti-D1.^41^ These findings conformed to an earlier study by Blank et al., showing that three peptides interfering with binding of monoclonal antibodies against domains 1 and 2, 3 and 4 prevented the development of experimental APS.^42^ In our study, neither IgA anti-β_2_GPI nor IgA anti-D1 associated with thrombotic or obstetric events among Sudanese patients. It is, however, difficult to determine whether this is a true negative finding or due to low statistical power. We are currently planning further investigations for IgA anti-domain 4/5 and IgG anti-D1 in Sudanese and Swedish cohorts.

IgA anti-β_2_GPI was found to be the most common isotype in SLE patients both in Sudan and Sweden using manufacturers’ cut-offs. In a recent paper investigating 526 Swedish SLE patients, and where our currently investigated Swedish patients were included, aPL cut-offs were adjusted to the 99th percentile value of 507 controls. IgA anti-β_2_GPI was found to be the most frequent aPL, irrespective of the co-occurrence of other isotypes, and significantly associated with non-Caucasian origin.^6^ In our current study, isolated IgA anti-β_2_GPI was not associated with any clinical event in Sudan or Sweden (data not shown). This is in contrast to an earlier study on 198 patients who tested positive for IgA anti-β_2_GPI recruited from three multi-ethnic cohorts where around 40% of participants were African Americans: the ‘Lupus in Minorities: Nature vs. Nurture’ (LUMINA) cohort, the Hopkins Lupus cohort and the Antiphospholipid Standardization laboratory cohort. In that study, isolated IgA antibodies were associated with arterial as well as any thromboses.^43^

Despite the inclusion of IgA aPL isotypes in the recent SLE classification criteria,^9^ there is yet no strong evidence relating these antibodies to thrombotic and/or pregnancy morbidity risk.^7^,^21^ We investigated aPL associations with APS-related clinical events using the common conventional and the adjusted cut-offs to the 95th percentiles of national controls. We determined the small number of Sudanese controls (*n* = 106) to be insufficient to determine the 99th percentile cut-offs accurately for such clinical comparisons,^44^ and used two independent ways to determine the 99th percentile for comparing the occurrence of aPL in Sudan and Sweden. For both countries, the 95th national cut-offs, compared to manufacturers’ cut-offs, showed more aPL associations to adverse pregnancy outcomes (Supplemental Tables S1 and S2). This might be of special interest, as it is concordant to previous reports where low-titre aPL (>95th and <99th) were clinically significant as risk factors for pregnancy morbidity in APS.^45^,^46^

Sudanese SLE patients had fewer venous thrombosis events compared to Swedish patients. However, when matching the cohorts for age and disease duration, this difference was lost. It is noteworthy that a patient history of arterial and/or venous thrombosis was not associated with any aPL in Sudanese patients, in contrast to Swedish patients. Nevertheless, most aPL associations with thrombosis were also lost in the matched Swedish patients, probably due to there being fewer subjects and thus a loss of statistical power. A new study presented at the 2019 ACR/ARP annual meeting, showed that African American SLE patients with venous thrombosis are 66% less likely to have a significant aPL profile compared to Caucasian patients.^47^ Therefore, negative aPL in African American SLE patients does not seem to exclude the risk of thrombosis. However, IgA aPL were not investigated in this study. The lack of an association of aPL with thrombosis in Sudanese patients might be in part due to the small number of events and/or aPL fluctuations over time.^48–50^ In our study, history of thrombosis was collected retrospectively from patients’ records. Levels of aPL have been previously shown to drop as a result of hydroxychloroquine (HCQ) treatment.^51^,^52^ In the current study, 9/15 (60%) Sudanese patients with thrombosis had ongoing treatment with HCQ compared to 22/77 (30%) Swedish patients (*p* = 0.02; data not shown). The common use of HCQ among Sudanese patients might also help to explain the lower prevalence of thrombosis compared to Swedish patients due to the preventive effect of this treatment.^53^

Limitations to this study are the cross-sectional approach where persistence of aPL was not investigated, and the limited number of Sudanese controls to calculate 99th percentile cut-offs accurately.

To the best of our knowledge, this is the first study designed to investigate aPL in SLE among Africans living in Northern Africa and also comparing to Caucasian populations using a similar methodological approach including national adjustment of reference ranges. The findings of high levels of IgA anti-β_2_GPI among Sudanese SLE patients as well as healthy controls compared to Swedes, together with the striking change in IgA aPL occurrence when adjusting to national reference ranges, have not been described before in patients of African origin. Our findings suggest that previous studies demonstrating a high IgA aPL prevalence might be re-evaluated using similar approaches.

## Supplemental Material

sj-pdf-1-lup-10.1177_0961203320945387 - Supplemental material for High IgA antiphospholipid autoantibodies in healthy Sudanese explain the increased prevalence among Sudanese compared to Swedish systemic lupus erythematosus patients Click here for additional data file. Supplemental material, sj-pdf-1-lup-10.1177_0961203320945387 for High IgA antiphospholipid autoantibodies in healthy Sudanese explain the increased prevalence among Sudanese compared to Swedish systemic lupus erythematosus patients by Sahwa Elbagir, Amir I Elshafie, Elnour M Elagib, NasrEldeen A Mohammed, Mawahib IE Aledrissy, Vivek Anand Manivel, Eleftheria Pertsinidou, Musa AM Nur, Iva Gunnarsson, Elisabet Svenungsson and Johan Rönnelid in Lupus
