# Supplementary material for: High IgA antiphospholipid autoantibodies in healthy Sudanese explain the increased prevalence among Sudanese compared to Swedish systemic lupus erythematosus patients
Source: Lupus. 2020 Aug 2;29(11):1412–22. doi: 10.1177/0961203320945387 (PMC7536526; doi:10.1177/0961203320945387)
Supplement: sj-pdf-1-lup-10.1177_0961203320945387 - Supplemental material for High IgA antiphospholipid autoantibodies in healthy Sudanese explain the increased prevalence among Sudanese compared to Swedish systemic lupus erythematosus patients [file sj-pdf-1-lup-10.1177_0961203320945387.pdf]

Supplementary table 1 Associations of antiphospholipid antibodies (aPL) to clinical events in 93 Sudanese SLE patients. Associations of aPL to APS-related events using manufacturers' cutoffs and the 95th percentile cutoffs of national controls. Data are demonstrated as number of patients with events among aPL positive patients (%)/number of patients with events among aPL negative patients (%). Number of patients investigated for aPL differed slightly and is given in the left column for the different autoantibodies. Thrombocytopenia at study inclusion was not included in this table (n=2). Significant p values are depicted in bold.

	any thrombotic n=10	P	venous thrombosis n=5	P	arterial thrombosis n=8	P	early miscarriage n=16	P	late miscarriage n=15	P	IUFD n=4	P	CVS n=6	P	ITP n=11	P
**FEIA**													1			
IgA CL common cutoff (n=92)	1(11.1%)/9(10.8%)	1	1(11.1%)/4(4.8%)	0.4	1(11.1%)/7(8.4%)	0.6	2(22.2%)/14(17.5%)	0.7	1(11.1%)/14(17.5%)	0.6	1(11.1%)/3(3.8%)	0.3	0(0)/6(7.3%)	1	0(0)/11(13.4%)	0.6
IgG CL common cutoff (n=92)	1(25%)/9(10.3%)	0.3	1(25%)/4(4.5%)	0.2	0(0)/8(9.1%)	1	0(0)/16(18.8%)	0.3	0(0)/15(17.6%)	0.3	0(0)/4(4.8%)	1	0(0)/6(6.8%)	1	1(25%)/10(11.5%)	0.4
IgM CL common cutoff (n=92)	1(33.3%)/9(10.1%)	0.2	1(33.3%)/4(4.5%)	0.1	1(33.3%)/7(7.9%)	0.2	0(0)/16(18.6%)	1	0(0)/15(17.4%)	1	0(0)/4(4.8%)	1	0(0)/6(6.7%)	1	1(33.3%)/10(11.4%)	0.3
IgA β2GPI common cutoff (n=92)	2(6.2%)/8(13.3%)	0.4	2(6.2%)/3(5%)	1	1(3.1%)/7(11.7%)	0.2	5(13.1%)/11(18.9%)	0.7	5(16.1%)/10(17.2%)	0.9	2(6.4%)/2(3.5%)	0.6	2(6.2%)/4(6.7%)	1	3(9.4%)8(13.6%)	0.7
IgG β2GPI common cutoff (n=92)	0(0)/10(12.2%)	0.3	0(0)/5(6.1%)	1	0(0)/8(9.7%)	1	3(30%)/13(16.5%)	0.3	1(10%)/14(17.7%)	0.5	0(0)/4(5.1%)	1	0(0)/6(7.3%)	1	2(20%)/9(11.1%)	0.4

IgM β2GPI common cutoff (n=92)	2(20%)/8(9.7%)	0.3	1(10%)/4(4.9%)	0.5	2(20%)/6(7.3%)	0.2	0(0)/16(20%)	0.1	2(22.2%)/13(16.2%)	0.6	1(11.1%)/3(3.8%)	0.3	3(30%)/3(3.7%)	**0.01**	2(20%)/9(11.1%)	0.4
IgA CL 95th cutoff (n=92)	1(11.1%)/9(10.8%)	1	1(11.1%)/4(4.8%)	0.4	1(11.1%)/7(8.4%)	0.6	2(22.2%)/14(17.5%)	0.7	1(11.1%)/14(17.5%)	1	1(11.1%)/3(3.8%)	0.3	0(0)/6(7.2%)	1	0(0)/11(13.4%)	0.6
IgG CL 95th cutoff (n=92)	1(12.5%)/9(10.7%)	0.9	1(12.5%)/4(4.8%)	0.4	0(0)/8(9.5%)	1	2(25%)/14(17.3%)	0.6	0(0)/15(18.5%)	0.3	0(0)/4(5.1%)	1	0(0)/6(7.1%)	1	1(12.5%)/10(12%)	1
IgM CL 95th cutoff (n=92)	1(12.5%)/9(10.7%)	0.9	1(12.5%)/4(4.8%)	0.4	1(12.5%)/7(8.3%)	0.7	0(0)/16(19.7%)	0.1	0(0)/15(18.5%)	0.3	1(12.5%)/3(3.7%)	0.2	1(12.5)/5(5.9%)	0.4	2(25%)/9(10.8%)	0.2
IgA β2GPI 95th cutoff (n=92)	2(11.1%)/8(10.8%)	1	2(11.1%)/3(4)	0.2	1(5.6%)/7(9.5%)	1	3(17.6%)/13(18.1%)	1	2(11.8%)/13(18.1%)	0.5	2(11.8%)/2(2.8%)	0.2	1(5.6%)/5(6.8%)	1	2(11.1%)/9(12.3%)	1
IgG β2GPI 95th cutoff (n=92)	0(0)/10(11.1%)	0.6	0(0)/5(5.6%)	1	0(0)/8(8.9%)	1	0(0)/16(18.4%)	1	1(50%)/14(16.1%)	0.2	0(0)/4(4.6%)	1	0(0)/6(6.7%)	1	0(0)/11(12.4%)	1
IgM β2GPI 95th cutoff (n=92)	2(14.3%)/8(10.3%)	0.5	1(7.1%)/4(5.1%)	0.6	2(14.3%)/6(7.7%)	0.3	2(15.4%)/14(18.4%)	1	3(23.1%)/12(15.8%)	0.5	1(7.7%)/3(4%)	0.5	3(21.4%)/3(3.8%)	**0.04**	2(14.3%)/9(11.7%)	0.8
**CIA**																
IgA B2GP1 D1 common cutoff (n=93)	1(16.7%)/9(10.3%)	0.6	1(16.7%)/4(4.6%)	0.3	1(16.7%)/7(8%)	0.5	1(16.7%)/15(17.9%)	0.9	0(0)/15(17.9%)	0.6	0(0)/4(4.8%)	1	0(0)/6(6.9%)	1	2(33.3%)/9(10.5%)	0.1

IgA B2GP1 D1 95th cutoff (n=93)	3(12%)/7(10.3%)	0.8	1(4%)/4(5.9%)	1	3(12%)/5(7.3%)	0.4	3(12.5%)/13(19.7%)	0.4	2(8.3%)/13(19.7%)	0.2	1(4.1%)/3(4.6%)	1	2(8%)/4(5.9%)	0.6	7(28%)/4(6%)	**0.004**
**PMAT**																
IgA CL common cutoff (n=93)	3(9.4%)/7(11.9%)	0.7	2(6.2%)/3(5.1%)	1	2(6.2%)/6(10.2%)	0.7	7(22.6%)/9(15.8%)	0.4	3(16.1%)/10(17.5%)	0.8	3(9.7%)/1(1.8%)	0.1	2(6.2%)/4(6.8%)	1	2(6.2%)/8(13.8%)	0.3
IgG CL common cutoff (n=91)	3(21.4%)/7(8.9%)	0.2	1(7.1%)/4(5.1%)	0.6	3(21.4%)/5(6.3%)	0.09	4(28.6%)/12(15.8%)	0.2	1(7.1%)/14(18.4%)	0.3	0(0)/4(5.3%)	1	3(21.4%)/3(3.8%)	**0.04**	3(21.4%)/8(10.3%)	0.2
IgM CL common cutoff (n=88)	2(20%)/7(9%)	0.3	1(10%)/3(3.9%)	0.4	2(20%)/6(7.7%)	0.2	4(40%)/12(16%)	0.07	3(30)/12(16)	0.3	2(20%)/2(2.7%)	0.07	2(20%)/4(5.1%)	0.1	2(20%)/9(11.7%)	0.4
IgA β2GPI common cutoff (n=93)	3(7.9%)/7(13.2%)	0.5	2(5.3%)/3(5.7%)	1	2(5.3%)/6(11.3%)	0.5	8(22.2%)/8(15.4%)	0.4	6(16.7%)/9(17.3%)	0.9	3(8.3%)/1(1.9%)	0.3	3(7.9%)/3(5.7%)	0.7	3(7.9%)/7(13.5%)	0.4
IgG β2GPI common cutoff (n=91)	0(0)/10(11.4%)	1	0(0)/5(5.7%)	1	0(0)/8(9.1%)	1	2(40%)/14(16.5%)	0.2	1(20)/14(16.5%)	1	0(0)/4(4.8%)	1	0(0)/6(6.8%)	1	1(20%)/10(11.5%)	0.5
IgM β2GPI common cutoff (n=88)	2(16.7%)/7(9.2%)	0.6	1(8.3%)/3(3.9%)	0.4	2(16.7%)/6(7.9%)	0.3	5(41.7%)/11(15.1)	**0.03**	3(25%)/12(16.4%)	0.5	2(16.7%)/2(2.8%)	0.09	2(16.7%)/4(5.3%)	0.2	3(25%)/8(10.7%)	0.1

IgA CL 95th cutoff (n=93)	3(8.3%)/7(12.7%)	0.5	2(5.6%)/3(5.4%)	1	2(5.6%)/6(10.9%)	0.5	9(25.7%)/7(13.2%)	0.1	5(14.3%)/10(18.9%)	0.6	1(8.6%)/1(1.9%)	0.3	2(5.6%)/4(7.3%)	1	2(5.5%)/8(14.8%)	0.3
IgG CL 95th cutoff (n=91)	3(12.5%)/7(10.1%)	0.7	1(4.2%)/4(5.8%)	1	3(12.5%)/5(7.2%)	0.4	5(21.7%)/11(16.4%)	0.6	4(17.4%)/11(16.4%)	0.9	1(4.3%)/3(4.5%)	1	3(12.5%)/3(4.3%)	0.2	3(12.5%)/8(11.7%)	1
IgM CL 95th cutoff (n=88)	3(18.7%)/6(8.3%)	0.3	1(6.2%)/3(4.2%)	0.5	3(18.7%)/5(6.9%)	0.1	7(43.7%)/9(13%)	**0.005**	3(18.7%)/12(17.4%)	0.9	2(12.5%)/2(2.9%)	0.2	3(18.7%)/3(4.2%)	0.07	3(12.5%)/9(12.7%)	1
IgA β2GPI 95th cutoff (n=93)	2(10%)/8(11.2%)	1	2(10%)/3(4.2%)	0.3	1(5%)/7(9.8%)	0.7	3(15.8%)/13(18.4%)	0.8	3(15.8%)/12(17.4%)	0.9	2(10.5%)/(2(2.9%)	0.2	0(0)/6(8.4%)	0.3	2(10%)/8(11.4%)	1
IgG β2GPI 95th cutoff (n=91)	2(10.5%)/8(10.8%)	0.9	1(5.3%)/4(5.4%)	1	2(10.5%)/6(8.1%)	0.7	5(26.3%)/11(15.5%)	0.3	3(15.8%)/12(16.9%)	0.9	0(0)/4(5.7%)	0.7	2(10.5%)/4(5.4%)	0.6	3(15.8%)/8(11%)	0.6
IgM β2GPI 95th cutoff (n=88)	2(10%)/7(10.3%)	0.9	1(5%)/3(4.4%)	1	2(10%)/6(8.8%)	1	7(35%)/9(13.8%)	**0.03**	3(15%)/12(18.5%)	0.7	3(15%)/1(1.5%)	**0.04**	3(15%)/3(4.4%)	0.1	3(15%)/8(11.9%)	0.7

Supplementary table 4 Associations of antiphospholipid antibodies (aPL) to clinical events in 333 Swedish SLE patients. Associations of aPL to APS-related events using manufacturers' cutoffs and the 95th percentile cutoffs of national controls. Data are demonstrated as number of patients with events among aPL positive patients (%)/number of patients with events among aPL negative patients (%). Number of patients investigated for aPL differed slightly and is given in the left column for the different autoantibodies. Significant p values are depicted in bold.

	any thrombotic n=76	P	venous thrombosis n=50	P	arterial thrombosis n=37	P	early miscarriage n=44	P	late miscarriage n=37	P	IUFD n=7	P	CVS n=33	P	Thrombocytopenia n=8	P
**FEIA**																
IgA CL common cutoff (n=291)	6(35.3%)/61(22.6%)	0.2	5(29.4%)/38(14.1%)	0.08	1(5.9%/33(12.2%)	0.4	4(25%)/35(14.5%)	0.2	4(25%)/33(13.6%)	0.2	2(14.3%)/5(2.2%)	0.06	1(5.9%)/29(10.6%)	0.5	2(11.8%)/4(1.5%)	**0.04**
IgG CL common cutoff (n=311)	9(42.8%)/64(22.5%)	**0.03**	6(28.7%)/41(14.3%)	0.08	3(14.3%)/33(11.5%)	0.7	4(25%)/39(15.2%)	0.3	3(18.7%)/34(13.2%)	0.5	2(13.3%)/5(2.1%)	0.06	5(23.8%)/26(9%)	**0.03**	2(9.5%)/5(1.7%)	0.07
IgM CL common cutoff (n=311)	5(35.7)/68(23.3%)	0.3	3(21.4%)/44(15%)	0.5	3(21.4%)/33(11.3%)	0.2	2(18.2%)/41(15.7%)	0.8	0(0)/37(14.1%)	0.2	0(0)/7(2.9%)	0.6	3(21.4%)/28(9.5%)	0.1	0(0)/7(2.4%)	1
IgA β2GPI common cutoff (n=288)	12(27.9%)/55(22.8%)	0.5	9(20.9%)/34(14.1%)	0.2	5(11.6%)/29(12%)	0.9	8(19.5%)/32(14.9%)	0.5	6(14.6%)/31(14.4%)	0.9	3(7.9%)/4(2%)	0.08	5(11.6%)/25(10.2%)	0.8	5(11.6%)/1(0.4%)	**0.0003**
IgG β2GPI	18(40.8%)/55(21%)	**0.004**	13(29.5%)/34(12.9%)	**0.005**	7(15.9%)/29(11%)	0.3	7(17.5%)/36(15.5%)	0.7	9(22.5%)/28(12%)	0.07	3(8.1%)/4(1.9%)	0.07	6(13.6%)/25(9.4%)	0.4	2(4.5%)/5(1.8%)	0.2

common cutoff (n=311)																
IgM β2GPI common cutoff (n=311)	8(29.6%)/65(23.3%)	0.5	6(22.2%)/41(14.6%)	0.3	4(14.8%)/32(11.4%)	0.6	4(16%)/39(15.8%)	0.9	1(4%)/36(14.5%)	0.1	0(0)/7(3.1%)	1	5(18.5%)/26(9.2%)	0.1	0(0)/7(2.5%)	1
IgA CL 95th cutoff (n=291)	35(22.7%)/14(25.9%)	0.6	8(14.8%)/35(15%)	0.9	7(13%)/27(11.6%)	0.8	10(19.6%)/29(14%)	0.3	7(13.7%)/30(14.4%)	0.9	4(8.3%)/3(1.6%)	**0.03**	6(11.1%)/24(10.2%)	0.8	4(7.4%)/2(0.8%)	**0.01**
IgG CL 95th cutoff (n=311)	15(35.7%)/58(22%)	0.05	12(28.6%)/35(13.2%)	**0.01**	6(14.3%)/30(11.3%)	0.6	10(28.6%)/33(13.9%)	**0.03**	9(25.7%)/28(11.8%)	**0.02**	3(8.6%)/4(1.9%)	0.06	8(19%)/23(8.6%)	**0.03**	3(7.1%)/4(1.5%)	0.05
IgM CL 95th cutoff (n=311)	10(25.6%)/63(23.6%)	0.8	6(13.4%)/41(15.3%)	0.9	5(12.8%)/31(11.5%)	0.8	6(18.2%)/37(15.5%)	0.7	4(11.7%)/33(13.8%)	0.7	2(6.4%)/5(2.3%)	0.2	6(15.4%)/25(9.2%)	0.2	0(0)/7(2.6%)	0.6
IgA β2GPI 95th cutoff (n=288)	26(26.3%)/41(22.1%)	0.4	21(21.2%)/22(11.9%)	**0.04**	10(10%)/24(13%)	0.5	16(18.4%)/24(14.3%)	0.4	12(13.8%)/25(14.8%)	0.8	6(7.2%)/1(0.6%)	**0.008**	11(11%)/19(10.2%)	0.8	5(5%)/1(0.5%)	**0.02**
IgG β2GPI 95th cutoff (n=311)	23(34.3%)/50(20.9%)	**0.02**	15(22.4%)/32(13.3%)	0.07	10(14.9%)/26(10.8%)	0.3	10(16.4%)/33(15.6%)	0.9	12(19.7%)/25(11.8)	0.1	3(5.4%)/4(2.1%)	0.2	9(13.4%)/22(9%)	0.3	4(6%)/(3(1.2%)	**0.04**
IgM β2GPI	17(29.8%)/56(22.5%)	0.2	13(22.8%)/34(!3.6%)	0.08	6(10.5%)/30(12%)	0.7	6(12.8%)/37(16.4%)	0.5	5(10.4%)/32(14.2%)	0.5	1(2.3%)/6(2.9%)	0.8	7(12.3%)/24(9.5%)	0.5	0(0)/8(2.8%)	0.3

95th cutoff (n=311)																
**CIA**																
IgA B2GP1 D1 common cutoff (n=333)	20(54%)/56(19.4%)	**<0.0001**	13(34.2%)/37(12.9%)	**0.0006**	9(23.7%)/27(9.4%)	**0.008**	6(19.3%)/38(15.6%)	0.6	3(9.7%)/33(13.5%)	0.5	2(6.7%)/5(2.2%)	0.2	6(15.8%)/27(9.3%)	0.2	1(2.6%)/7(2.4%)	0.9
IgA B2GP1 D1 95th cutoff (n=333)	22(32.8%)/54(20.9%)	**0.04**	14(20.6%)/36(13.9%)	0.2	10(14.7%)/26(10.1%)	0.3	10(18.5%)/34(15.4%)	0.6	6(11.1%)/30(13.6%)	0.6	4(8%)/3(1.5%)	**0.03**	9(13.2%)/24(9.2%)	0.3	4(5.9%)/4(1.5%)	0.06
**PMAT**																
IgA CL common cutoff (n=331)	25(33.7%)/51(20.5%)	**0.01**	19(25.3%)/31(12.4%)	**0.007**	11(14.7%)/25(10%)	0.3	8(13.1%)/36(17.1%)	0.5	7(11.5%)/29(13.7%)	0.6	3(5.3%)/4(2.1%)	0.2	9(12%)/24(9.5%)	0.5	6(8.3%)/2(0.8%)	**0.002**
IgG CL common cutoff (n=326)	26(36.1%)/49(19.9%)	**0.004**	22(30.6%)/27(10.9%)	**<0.0001**	8(11.1%)/28(11.3%)	0.9	12(19.7%)/31(14.9%)	0.4	11(18%)/25(12%)	0.2	3(5.4%)/4(2.1%)	0.2	8(11.1%)/25(9.9%)	0.8	6(8.3%)/2(0.8%)	**0.002**
IgM CL common cutoff (n=327)	10(23.8%)/63(22.7%)	0.8	8(19%)/39(14%)	0.4	4(9.5%)/31(11.1%)	0.7	6(16.7%)/36(15.4%)	0.8	4(10.8%)/32(13.7%)	0.6	0(0)/7(3.2%)	0.6	6(14.3%)/26(9.2%)	0.3	0(0)/8(2.9%)	0.6

IgA β2GPI common cutoff (n=331)	24(32%)/52(21%)	0.05	19(25%)/31(12.5%)	**0.009**	9(11.8%)/27(10.9%)	0.8	10(16.1%)/34(16.3%)	0.9	8(12.9%)/28(13.3%)	0.9	4(6.8%)/3(1.6%)	0.06	8(10.5%)/25(10%)	0.9	4(5.3%)/4(1.6%)	0.09
IgG β2GPI common cutoff (n=326)	20(40%)/55(20.5%)	**0.003**	16(32%)/33(12.3%)	**0.0004**	6(12%)/30(11.1%)	0.9	7(16.7%)/36(15.9%)	0.9	9(21.4%)/27(11.8%)	0.09	3(7.9%)/4(1.9%)	0.07	6(12%)/27(9.9%)	0.6	4(8%)/4(1.5%)	**0.02**
IgM β2GPI common cutoff (n=326)	11(26.8%)/62(22.4%)	0.5	9(21.9%)/38(13.7%)	0.2	3(7.3%)/23(11.5%)	0.4	5(14.7%)/37(15.8%)	0.9	3(8.6%)/33(14.1%)	0.4	0(0)/7(3.2%)	1	5(12.2%)/27(9.6%)	0.6	0(0)/8(2.9%)	0.6
IgA CL 95th cutoff (n=331)	33(28%)/43(21%)	0.1	24(20.2%)/26(12.7%)	0.07	14(11.8%)/22(10.7%)	1	13(13.7%)/31(17.5%)	0.4	10(10.5%)/26(14.6%)	0.3	5(5.6%)/2(1.2%)	0.1	11(9.2%)/22(10.6%)	0.7	6(5%)/2(0.9%)	**0.03**
IgG CL 95th cutoff (n=326)	33(32%)/42(19.5%)	**0.01**	25(24.3%)/24(11.1%)	**0.002**	13(12.6%)/23(10.6%)	0.6	13(14.6%)/30(16.7%)	0.7	14(15.7%)/22(12.1%)	0.4	3(3.8%)/4(2.4%)	0.7	12(11.6%)/21 (9.5%)	0.6	6(5.8%)/2(0.9%)	**0.01**
IgM CL 95th cutoff (n=327)	12(26.1%)/61(22.3%)	0.6	10(21.7%)/37(13.5%)	0.1	4(8.7%)/31(11.3%)	0.6	6(16.2%)/36(15.5%)	0.9	4(10.5%)/32(13.8%)	0.6	0(0)/7(3.2%)	0.6	6(13%)/26(9.3%)	0.4	0(0)/8(2.9%)	0.6
IgA β2GPI 95th cutoff	33(26.8%)/43(21.5%)	0.3	23(18.5%)/27(13.5%)	0.2	15(12%)/21(10.5%)	0.6	13(13%)/31(18%)	0.3	11(11%)/25(14.4%)	0.5	6(6.4%)/1(0.6%)	**0.01**	12(9.7%)/21(10.3%)	0.8	6(4.8%)/2(1%)	0.06

(n=331)																
IgG β2GPI 95th cutoff (n=326)	28(33.7%)/47(20%)	**0.01**	22(26.5%)/27(11.4%)	**0.001**	10(12%)/26(11%)	0.8	11(15.7%)/32(16.1%)	0.9	13(18.6%)/23(11.5%)	0.1	3(4.8%)/4(2.2%)	0.4	9(10.8%)/24(10%)	0.8	7(8.4%)/1(0.4%)	**0.0004**
IgM β2GPI 95th cutoff (n=326)	15(26.8%)/58(22.1%)	0.4	13(23.2%)/34(12.9%)	**0.048**	3(5.4%)/32(12.2%)	0.1	7(15.2%)/35(15.8%)	0.9	5(10.6%)/31(13.9%)	0.5	1(2.4%)/6(2.9%)	0.9	5(8.9%)/27(10.1%)	0.8	1(1.8%)/7(2.6%)	0.7
